# Metabolomics in archaeological science: A review of their advances and present requirements

**DOI:** 10.1126/sciadv.adh0485

**Published:** 2023-08-11

**Authors:** Diego Badillo-Sanchez, Maria Serrano Ruber, Anna Davies-Barrett, Donald J. L. Jones, Martin Hansen, Sarah Inskip

**Affiliations:** ^1^ School of Archaeology and Ancient History, University of Leicester, Leicester, UK.; ^2^ Leicester Cancer Research Centre, RKCSB, University of Leicester, Leicester, UK.; ^3^ The Leicester van Geest MultiOmics Facility, University of Leicester, Leicester, UK.; ^4^ Environmental Metabolomics Lab, Department of Environmental Science, Aarhus University, Roskilde, Denmark.

## Abstract

Metabolomics, the study of metabolites (small molecules of <1500 daltons), has been posited as a potential tool to explore the past in a comparable manner to other omics, e.g., genomics or proteomics. Archaeologists have used metabolomic approaches for a decade or so, mainly applied to organic residues adhering to archaeological materials. Because of advances in sensitivity, resolution, and the increased availability of different analytical platforms, combined with the low mass/volume required for analysis, metabolomics is now becoming a more feasible choice in the archaeological sector. Additional approaches, as presented by our group, show the versatility of metabolomics as a source of knowledge about the human past when using human osteoarchaeological remains. There is tremendous potential for metabolomics within archaeology, but further efforts are required to position it as a routine technique.

## Teaser

Archaeological scientists have applied metabolomics, producing key developments, but difficulties persist in its implementation.

## INTRODUCTION

Archaeological science uses modern analytical tools to investigate surviving materials from the past. Various methods, including spectroscopic, spectrometric, isotopic, molecular, and genetic techniques, have been adapted from other fields and applied to archaeological remains (*1*). Using these tools, archaeological materials can be broadly split into two groups (*2*). The first includes objects such as tools, weapons, or containers that were in contact with organic molecules, which are preserved as residues and combusted remains or introduced as part of the object’s composition during its manufacturing process (e.g., glues). The second group includes direct human, animal, or plant remains, which inherently contain biological data. Such residues and remains have the potential to yield information using biomolecular approaches. However, physical-chemical transformation/alteration by taphonomic processes, including degradation and environmental exposure, can alter biological information in both types of archaeological remains over time before discovery. In addition, alterations in the composition of the material during and after storage can also occur (*3*–*6*). Despite these factors, positive results from investigations of elemental and macromolecular composition suggest that the study of biomolecules remains a viable approach in archaeology.

Among the biomolecular techniques widely accepted and used in archaeology today are “omics” approaches (e.g., genomics, proteomics, and metabolomics). These are analytical tools developed and used in a variety of scientific fields. Genomics and proteomics have been widely adopted in archaeology and have significantly changed paradigms of the past, but metabolomics has been slow to gain traction (*7*). Metabolomics is a technique that analyzes metabolites: Small molecules (with a molecular weight lower than 1500 Da) formed when an organism breaks down food, chemicals, or its own tissue and are recovered from biofluids, cells, and tissues (*8*, *9*). It was initially used to study fresh tissue/fluids in living organisms to capture specific metabolic events, revealing information about the phenotype of a tissue, organism, or population of interest. This approach allows for the study of humans, microorganisms, animals, or plants and can be used to assess various outcomes, such as metabolomic profiles, understanding of metabolic pathways, discovery of biomarkers for specific conditions, or evaluation of phenotypic differences between populations (*9*, *10*). Such investigations can offer insights into the impact of external factors, physiological changes, and pathophysiology on an organism’s metabolic status (*11*). This information can be used to evaluate differences between populations or groups subject to different stressors and has broad applications in the medical, environmental, pharmaceutical, and food sectors (*9*).

Advances in analytical instrumentation and computer processing (including the development of new software tools) have made metabolomics a routine approach for characterizing molecules in life sciences. Increased options for analytical experimentation and method validation are allowing for greater control of experimental design, confounders, and sample size and replicability. Metabolomic approaches are now being adapted for use in other fields, including archaeology. This review presents the current state of the art of metabolomics in archaeological science and its potential as a tool within the field while exploring the perspectives/disciplines from which metabolomics was adopted. In addition, it discusses the limitations and further requirements of an archaeometabolomic approach (use of metabolomics to study archaeological materials and its metadata) to allow for its routine incorporation into an archaeological science toolkit. For this to happen, research is necessary to expand our knowledge of the composition of past materials and to understand the effects of taphonomy on long-buried objects. This will enable us to develop optimal protocols before their routine use in archaeology (*1*, *2*, *12*). If these issues can be addressed, archaeometabolomics could provide valuable insights into the impact of environmental, socioeconomic, and cultural changes; dietary shifts; population expansion and conflict; manufacturing processes; human-animal relationships; and other important variables within different past human populations.

### Metabolomics: Generalities

**Fig. 1.**
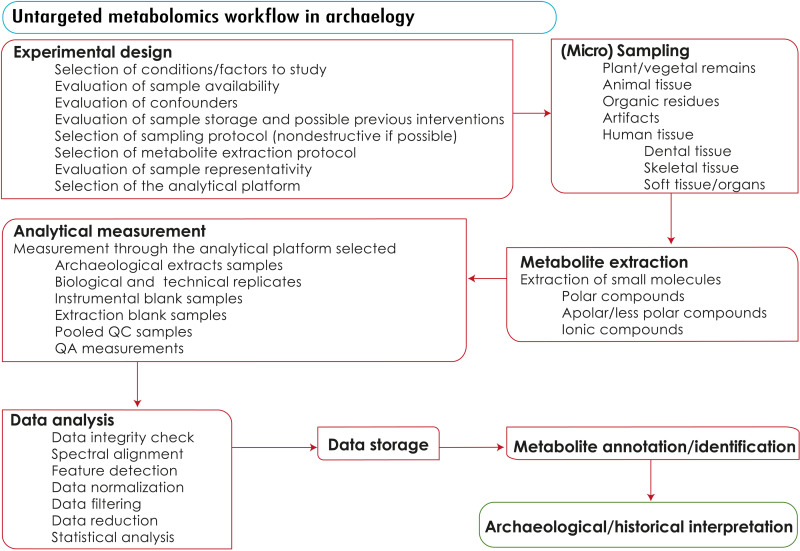
Metabolomics workflow performed in archaeological science. Commonly used sequence of steps involved in the use of metabolomics for the study of archaeological materials to achieve an optimal archaeological/historical interpretation.

Metabolomic studies can be performed using untargeted or targeted strategies, each with their own advantages and limitations (*13*). Untargeted metabolomics analyzes all detectable metabolites in a sample (*14*), while targeted metabolomics measures defined groups of metabolites with prior knowledge of the molecule(s) of interest (*15*). Metabolomic workflows typically involve several steps, including experimental design, sample selection, preparation, data collection, processing, statistical analysis, and interpretation/validation of biomarkers (*16*) (see Fig. 1). A study’s quality depends on robust experimental design, quality control (QC)/quality assurance (QA), and thorough consideration of confounders and variability to ensure that the data accurately represents biological information from the individuals/samples (*17*). Archaeologists have begun adopting metabolomic techniques from other fields to develop their own extraction and measurement protocols for archaeological research. However, additional precautions must be taken, as the initial information and metadata available for samples may be limited or nonexistent, and exogenous contaminants may interfere with the biological information retrieved. Furthermore, the impact of various taphonomic processes requires additional efforts to achieve results comparable with those obtained from the study of living organisms.

Metabolomic approaches aim to monitor changes in a large and diverse array of molecules. To do this, an analytical platform of coupled apparatus is used to separate and detect small molecules present in the matrix under investigation (*18*–*22*). Separation techniques include but are not limited to gas chromatography (GC), liquid chromatography (LC), and capillary electrophoresis; meanwhile, detection instrumentation can involve the use of mass spectrometry (MS), nuclear magnetic resonance, infrared spectroscopy, and ultraviolet spectroscopy, among others. The complexity of biological samples means that it is now impossible for any single analytical platform to cover the full metabolome (*23*). Chemical properties such as hydrophobicity, volatility, and polarity are variable among molecules. Thus, GC-MS is preferred for nonpolar volatile molecules, reversed-phase LC coupled with MS has been used for analyzing nonpolar to polar molecules, and hydrophilic interaction LC coupled to MS is preferred for the analysis of polar metabolites. There is a substantial overlap between these techniques, which leads to a comprehensive analysis when all three are used (*24*). Thus, a series of instruments and protocols must be used to achieve the best metabolite coverage and understanding of the phenomena of interest (*13*, *25*). Despite the differences between platforms, deproteinized samples are preferred, as proteins can significantly affect precision, accuracy, and instrument life span (*26*, *27*).

Measurement in metabolomics can be either direct (from the sample matrix) or indirect (from a solution produced from chemical extraction). Direct measurement approaches are less common than extraction methods because they require samples that are compatible with the analytical platform in terms of size, shape, and chemical composition. Indirect measurements require reliable, reproducible, and standardized extraction steps to isolate the biological information of interest from the samples. The extent of recovery of different metabolite classes after extraction depends on the solvent or solvent mixtures and ratios used (*28*). In addition, it is critical that the resultant matrices do not present interferences with the platform to be used, as for example, spectrometric aberrations or incompatibilities with the ionization source in the case of LC-MS (*28*).

### Metabolomics using fresh tissues: Establishing a baseline for archaeometabolomics

Attempts to standardize and optimize metabolomic approaches in fresh tissue have been taking place for over 20 years. This has led to the development of numerous libraries, databases, and standards for metabolomic identification in various disciplines (*29*–*31*). Human metabolomic studies use different fresh sample types including cells; saliva; urine; blood; serum; feces; and heart, skeletal, muscle, kidney, liver, skin, and brain tissues (*32*–*34*).

Metabolomics is becoming increasingly important for fundamental research into human disease mechanisms and epidemiology. Studies have reported results for health conditions including cardiovascular events, diabetes, fatty liver and nonalcoholic steatohepatitis, insulin resistance, pancreatic islet biology, oral cancers, and osteoporosis (*33*–*40*). Further targeted metabolomic analysis has been used to study the exposome in the dental tissue of deciduous teeth (*41*). Metabolomic studies of plants, fungi, bacteria, and animals have also been conducted, with important health information extracted from bone tissue in animal studies (*42*–*44*). Metabolomics has also found significant application in the food industry for determining chemical profiles that allow for the identification of product adulteration, which remains detectable even after processing (*45*, *46*).

### Metabolomics in forensics: A broader horizon

Metabolomic analysis of nonliving organisms, such as those found on archaeological sites, is challenging due to degradation, necrosis, and microcellular imbalances that facilitate colonization by different microorganisms, which, in turn, produce extraneous metabolites. These processes can complicate interpretation of results, particularly with respect to metabolites produced by foreign microbes (*5*, *47*). However, forensic investigations using metabolomics have demonstrated that it is possible to extract biological information associated with an individual even after necrotic and postmortem processes have occurred (*48*, *49*). Forensic scientists have collected a large variety of samples from recently deceased individuals and have shown that metabolomic workflows can be performed in similar ways to those used in fresh tissue studies. Analysis of blood, plasma, urine, and brain tissue from forensic contexts has been used to explore questions surrounding the life and death of humans and animals, such as postmortem interval, toxicology, and decay (*48*–*50*).

Such metabolomic analysis provides a snapshot of the sample at the time of collection, with the proviso that death acts as a “quenching mechanism.” This stops the different intrinsic metabolomic processes occurring while the individual was alive, but this does not halt the production of extrinsic metabolites (which result from the interaction between the postmortem body and its environment). This permits an investigation of the metabolites from postmortem processes, as well as those indicating the biological condition of the individual before death. This is relevant to archaeology in two ways. First, forensic studies demonstrate that metabolites from an individual’s living state may still be present despite degradation processes affecting composition and concentration. Second, it shows that metabolites can provide information about postmortem funerary and depositional practices.

### Current state of art of metabolomics in archaeology

In the past decade, metabolomics has been used by archaeologists to explore a variety of questions using unconventional materials. For example, archaeological scientists have identified the potential of using metabolomics to complement residue analysis in archaeological materials. Although highly altered by postdepositional processes, the chemical composition of biological archaeological remains can retain some metabolites comparable to metabolomic profiles constructed from fresh tissue (*48*). If such metabolomic profiles can be constructed reliably, then they offer potential high-resolution indicators of past conditions or states. This approach can, for example, help archaeologists investigate ancient recipes, interpret the dietary behavior of ancient populations, and assess the availability of food in the past. Successful archaeological metabolomics studies have used metabolomic approaches to study samples of archaeological wine, tobacco, silk, beeswax, culinary residues, inks, dung, human dental calculus, and mummified human remains. However, the success of such studies is limited by the number of individuals available per study, which are themselves affected by the number of artifacts/remains recovered, sample availability, and collection access, posing a significant limitation that can be challenging to overcome.

Some archaeological researchers have adapted metabolomic statistical workflows originally devised for analysis of metabolomic results from living tissue and forensic studies. For example, López-García *et al.* (*51*) applied partial least squares discriminant analysis, a statistical tool commonly used in metabolomics, to more than 100 ceramics from the site of Xalasco, Mexico, using nondestructive energy-dispersive x-ray fluorescence spectroscopy. The study identified compositional outliers and statistical clustering that allowed researchers to classify and discriminate between two similar pottery groups, which were interpreted as different recipes for pottery production.

**Fig. 2.**
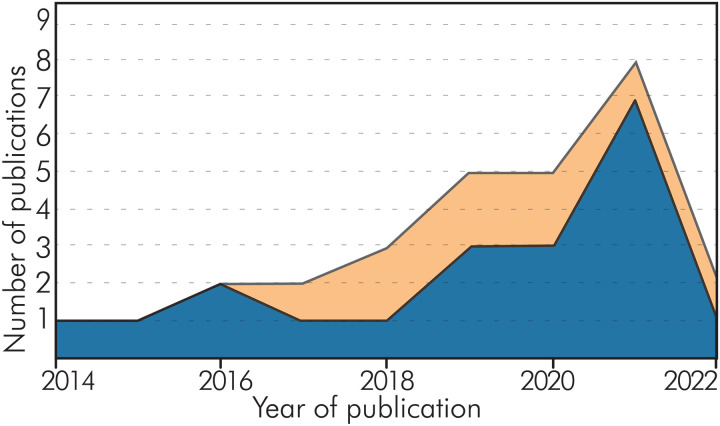
Publications available in digital repositories between 2014 and 2022 related to archaeometabolomics. Diagram showing the variation with respect to the use of a complete metabolomics strategy in the research (blue) and those that followed partially or sections of the metabolomics workflow (orange).

Figure 2 demonstrates the increase in the use of metabolomic approaches in archaeology since 2014, although they remain scarce. Table 1 shows the limited number of hits on principal search engines for terms such as “metabolomics” and “archaeology” as well as “metabolomics” and “archaeometry,” which highlights the deficit of metabolomic work in archaeology compared to other fields. These sparse numbers indicate that there are some difficulties in implementing metabolomics as a routinely used tool in the discipline, suggesting that the application of the method in archaeology requires further research.

**Table 1.** Variation of publications for metabolomics and its use in forensics, archaeology, and archaeometry. Results of total hits were obtained after searching in three of the most used scholar search engines for metabolomics terms in the period 2010 to 2022.

Search terms	Google Scholar	Web of Science	ScienceDirect
“metabolomics”	178,000*	42,625*	45,409*
	46,400†	4,896†	9,250†
“metabolomics” and “forensic”	11,500*	149*	1,167*
	2,020†	32†	284†
“metabolomics” and “archaeology”	1,190*	5*	249*
	194†	1†	49†
“metabolomics” and “archaeometry”	121*	0*	28*
	20†	0†	1†

*Total of manuscript hits.

†Total of manuscripts as review.

### Archaeometabolomics and other omics

Metabolomics has been combined with other omic techniques to enhance results in archaeological studies. For example, Szulc *et al.* combined metabolomics and metagenomics to investigate historical beeswax seals (*52*) and silk (*53*) that had degraded over time. The studies detected differences in the chemical profile of archaeological materials compared to control samples, identifying hundreds of metabolites composed mainly of different peptides, amino acids, and their derivatives. The combination of DNA sequencing and the characterization of metabolites through metabolomics allowed for the identification of microbes that attack organic material. This helped to differentiate between historical and control samples and distinguish between microbiological and intrinsic artifact metabolites.

Similarly, metabolomics and proteomics have also been combined. Zilberstein *et al.* (*54*) used a noninvasive sampling method to assess the binder used in ink from a scroll manuscript found near the Dead Sea. The researchers characterized the composition of the binder, discovering the use of a mixture of plant proteins, glycoproteins, plant acids, and terpenes. The protein and metabolomic composition allowed the researchers to identify the use of arabic gum from two tree species commonly found in the Middle East, where the scrolls were located.

These studies establish the potential for combining metabolomics with other techniques to expand the knowledge of artifact composition and manufacturing procedures of the past, thus answering questions about cultural heritage and biodeterioration. However, archaeological materials present barriers to scientific study due to limitations in the quantity and type of sample available. To overcome this challenge, it may be most appropriate to apply multiple analytical techniques, including metabolomics, to the same sample if possible, limiting the destruction of unique archaeological materials. Manfredi *et al.* (*55*) have developed a noninvasive sampling strategy for archaeological and artistic materials, using a plastic film with ion exchange and resins to extract both proteins and small molecules. This method is efficient and leaves no trace on the surface of the source material, maintaining the integrity of the sampled object and allowing for the possibility of further future sampling.

### Archaeometabolomics of plants, foods, and intoxicants

Metabolomic approaches can be used for identifying preserved plants and determining related information, such as provenance, stress regulators, and cultivation practices. For instance, Righetti *et al.* (*56*) used metabolomics to assess different ancient *Triticum* varieties, important not only for understanding past cultivation practices but also for their potential suitability as sustainable food today. Similar approaches have also been used to identify the origin of wooden objects, demonstrating the potential of metabolomics for provenancing archaeological items. Creydt *et al.* (*57*) used tandem LC-MS to differentiate the geographic origin of 10 wood samples from Belize and 11 samples from Suriname. They found that metabolomics was a more reliable approach for sourcing the origin of wood than genetics or stable isotopes, particularly for samples that had undergone significant decay while buried. Decq *et al.* (*58*) used a nontargeted approach to discriminate between four different plant resins used to coat historic lacquered objects by identifying a wide range of biomarkers, producing a large library of resin markers for future studies.

A number of archaeometabolomic studies have enhanced our understanding of past food consumption by analyzing various organic residues found in artifacts. For example, Garnier and Vedeler (*59*) identified markers of alcoholic fermentation, fruits, and fish in medieval soapstone vessels from southern Norway, distinguishing between animal and vegetal residues. Manzano *et al.* (*60*) used a multiplatform approach to study organic residues in prehistoric pottery from a Spanish site, identifying evidence of past human food production through distinctive chemical fingerprints such as unsaturated fatty acids and short-chain fatty acids. Work by Farag *et al.* (*61*) used a targeted approach to provide insight into ancient brewing methods and beer composition by analyzing residues recovered from vats from an Egyptian archaeological site. In addition, Roullier-Gall *et al.* (*62*) traced the chemical fingerprint of an historic bottle of wine using metabolomics to assess its age and grape variety.

Demonstrated by the number of studies undertaken on alcoholic beverages, one area of particular interest is the use of intoxicants. Archaeologists are increasingly using both targeted and untargeted metabolomic approaches to study organic residues and identify intoxicants consumed in the past, such as wine and tobacco. While previous attempts at identifying tobacco use have focused on the targeted identification of nicotine through GC-MS (*63*, *64*), recent studies such as those by Damitio *et al.* (*65*), Tolan *et al.* (*66*), Brownstein *et al.* (*67*), and Zimmermann *et al.* (*68*) have used different analytical platforms and strategies to identify a greater variety of tobacco compounds and discriminate to the species level. These approaches have allowed researchers to ask more complex questions about plant use in the past and gain a better understanding of the consumption of intoxicants.

### Archaeometabolomics of animal and human remains

In addition to looking at plants and their products, metabolomics is applied to human, and animal remains provide direct insight into our ancestors and their interactions with fauna, offering unique data that can address research questions related to health and the human condition. Metabolomics can be used to study extinct animals, as demonstrated in the work of Kostyukevich *et al.* (*69*) on mammoths. Sex hormones and bile acids in Neolithic animal dung layers recovered from a cave in El Mirador (Sierra de Atapuerca, Burgos, Spain) were analyzed using metabolomics to ascertain the ways in which the cave was used as a sheepfold (*70*). Other metabolomic approaches have also enhanced our understanding of past funerary practices, as in the study by Brockbals *et al.* (*71*) on canopic jars and samples from mummified persons from Egypt, which identified embalming ingredients and materials not previously known from the literature.

Within bioarchaeology, multiomic approaches have been used to study human remains, as highlighted by Wright *et al.* (*72*) in a review of multiomic investigations of calcified dental plaque (calculus) from archaeological remains. By using DNA sequencing, metabolomics, and proteomics applied to calculus, it is possible to identify oral microorganisms and their relationship to periodontal disease in ancient populations (*73*). Sørensen *et al.* (*74*) developed an analytical method for identifying drugs and their metabolites in human dental calculus for prospective application to archaeological individuals. This method was evaluated on calculus from forensic contexts and found that drugs were detected in this material, providing results comparable to those obtained from blood studies.

While metabolomics has been used to explore different human and animal tissues, archaeological human skeletal remains (osteoarchaeological material) have only recently started to be explored by our group at the University of Leicester.

### Our approach to archaeometabolomics

Our group, working on the “Tobacco, Health and History” project, has initiated an approach to improve the application of metabolomics in archaeology. Using an ultraperformance LC coupled to high-resolution MS platform, we have investigated human osteoarcheological material and demonstrated that it preserves many small molecules that can be relevant to the study of the past through a systematic metabolomics workflow (*75*). Although archaeological bones contain lower concentrations of metabolites compared to fresh tissue, they still exhibit a diverse range of polar and less polar/apolar molecules. This shows that archaeological human skeletal remains are a feasible and accessible source of biological information to study the human condition in previous periods of time. In addition, we have shown that metabolomics can be used to study ancient dry botanical material to increase our understanding of plant use and exploitation in the past (*76*).

### The future of archaeometabolomics: Challenges and prospects

Metabolomics has become a reliable technique with advanced analytical platforms, standardized protocols, and publicly available repositories. However, a paucity of studies using metabolomics in archaeological science has led to a delay in the development of archaeology-specific protocols, libraries, and databases, in turn, resulting in higher costs and hindering integration with other archaeological tools. It is evident that the use of metabolomics within archaeology is still in its initial stages and requires improvements from various perspectives.

First, it is important to acknowledge that archaeological matrices differ from fresh tissues, and archaeological scientists need to understand their own unique requirements before using metabolomic approaches. It is important to have detailed knowledge of the material, especially relevant taphonomic processes, and the archaeological context. The type of archaeological material used should be carefully considered to control the different degradation processes and intrinsic variability. This requires a series of steps, including exploring the archaeological context, collecting metadata, selecting appropriate sampling and extraction processes, accounting for confounders, characterizing the sample’s biological and nonbiological composition, and validating the analytical steps in the workflow.

### Technical challenges

There are numerous technical challenges in metabolomics, including the use of multiple computational tools to process data after analytical measurements. Now, there is no consensus on a standardized data pipeline, resulting in varied processing steps depending on the platform or research group handling the data. This lack of standardization can compromise robust reproducibility between platforms or laboratories, especially for researchers entering into metabolomics from nontraditional fields like archaeology (*77*). Researchers have made significant improvements in addressing the needs of various sectors for solving analytical, research, and practical questions. These advances can be adapted from other disciplines, such as the improvements in platforms over the past few decades (*78*), scientific workflow managers from medical sciences, and data processing pipelines constructed with flexible scripts in, for example, R and Python ecosystems (*79*). However, archaeological science requires specific solutions to address its unique challenges, rather than simply adapting existing metabolomic advances. To achieve comparable results to other fields with longer experience in this field, greater efforts are needed to develop protocols, repositories, and metabolite standards (*80*), accompanied by better analytical and computational approaches for solving the challenges faced in archaeological science.

Archaeological adaptation of metabolomics presents both technical and administrative challenges that need to be resolved to allow its regular use in the field. To overcome initial obstacles, better communication between researchers and institutions that curate archaeological remains is needed to showcase the benefits of using metabolomics and promote more sources for research. Trained professionals familiar with archaeological materials are also necessary to ensure proper interpretation of findings. Furthermore, it will be necessary to produce archaeo-specialist informaticians and data handling experts. This is critical to large-scale analysis.

### QC and QA

Metabolomics in archaeology requires workflow controls and assurance steps to check platform performance, ensure data, and result quality, as in any other field. When studying archaeological materials, QA and QC steps are crucial. Archaeological materials often have high variance and limited metadata, and the concentration of small molecules can be extremely low (in contrast to the elevated number and concentration of metabolites in life sciences studies). Therefore, QA procedures and data treatment are necessary to preserve biological information and remove extraneous signals and nonbiological information that can be introduced during the metabolomic assay.

Among the different challenges in the use of metabolomics in the study of archaeological materials, one singular aspect sometimes underestimated is the use of “pooled QC” samples for the QA procedures. Pooled QC samples combine aliquots from all the different samples used within the study to create multiple replicates of a similar composition to the material being tested. The inclusion of pooled QC samples distributed among an analytical batch ensures that the resultant data and any operations during the metabolomics workflow are not altering the biological information produced from the samples (*81*, *82*). Pooled QC samples have been used in combination with informatics tools in living studies to solve issues that can arise, such as batch errors in the metabolomic assays (*83*).

Every instrument can exhibit instrumental drift; thus, blank and pooled samples and randomized measurements are mandatory controls in all metabolomic assays (*17*, *84*). However, because of the low volume/mass of samples and low volume extraction in archaeological material, it may not always be possible to obtain large volumes of pooled samples from archaeological materials for repeated injections or for later corrections. To address this issue, it is crucial for archaeologists to consider how much material they can use, with the knowledge that it is necessary to use pooled QC samples even if they cannot be injected as frequently as in living studies. Failure to do so could have a significant impact on the validity of results and security of conclusions drawn. This has ethical implications, because archaeological material may be scarce and is often irreplaceable. Furthermore, because of the extensive use of computational tools in the analysis of high-volume data associated with metabolomic studies, standardized biostatistic steps must be performed to ensure the integrity, security, accuracy, and reliability of all outputs (e.g., data, results, and inference) (*85*, *86*).

### Compound identification

Compound identification is a complex problem in metabolomics that persists in LC-MS measurements. This is due to changes in response according to the analytical platform and protocol used, as well as the inherent resolution of instruments, which do not always allow for easy discrimination between molecules. For example, with an instrument resolution of c. 250 Da, there will be around 1500 possible identifications for a single molecule (*87*, *88*). This problem is particularly relevant in archaeology, as databases and chemical standards are not extensive enough to allow easy comparison of altered matrices, such as those that have undergone curation or decay. To address this issue, it is necessary to increase the number of studies and expand existing repositories, libraries, and databases. By using different analytical platforms, a broader range of molecules in archaeological material can be explored. These markers can then be added to archaeology-specific metabolomic libraries to improve the future interpretation of complex mixtures in historical/archaeological objects.

Using multiple and orthogonal analytical platforms to obtain a comprehensive metabolomic coverage of a sample is a major challenge in archaeology due to limited sample sizes and volume, as well as legal/ethical concerns. The cost of using the infrastructures of other institutions for such studies also increases with the number of measurements performed, making it difficult to experiment with unfamiliar protocols. Therefore, archaeologists should focus on fundamental research to determine the best extraction processes, protocols, and platforms for different materials and to avoid wasting research funds and precious materials. In the future, dedicated instruments and centers for archaeological proteo-metabolomics will be necessary, which can only be achieved if there is sufficient demand to lower infrastructure and personnel costs.

## CONCLUSIONS

More activities are needed to advance metabolomics in archaeology, but the potential impact of the approach on our understanding of humanity is significant. If current issues are tackled, then archaeometabolomics will improve knowledge of past human health conditions and diets, as well as elucidating the influence of changing environmental factors and other differences among ancient populations. In addition, metabolomic analysis of archaeological materials will help to reveal ancient recipes, customs, social practices, and manufacturing processes, while studying faunal and plant remains will help in the investigation of economic changes, human and animal interactions, plant use as intoxicants, anthropogenic selection, and the impact of selective breeding of both plants and animals. All of these will expand our knowledge of the human past and answer new research questions integral to understanding our history.
